# E-GWAS: an ensemble-like GWAS strategy that provides effective control over false positive rates without decreasing true positives

**DOI:** 10.1186/s12711-023-00820-3

**Published:** 2023-07-05

**Authors:** Guang-Liang Zhou, Fang-Jun Xu, Jia-Kun Qiao, Zhao-Xuan Che, Tao Xiang, Xiao-Lei Liu, Xin-Yun Li, Shu-Hong Zhao, Meng-Jin Zhu

**Affiliations:** 1 Key Lab of Agricultural Animal Genetics, Breeding, and Reproduction of Ministry of Education, Huazhong Agricultural University, Wuhan, 430070 China; 2 The Cooperative Innovation Center for Sustainable Pig Production, Huazhong Agricultural University, Wuhan, 430070 China

## Abstract

**Background:**

Genome-wide association studies (GWAS) are an effective way to explore genotype–phenotype associations in humans, animals, and plants. Various GWAS methods have been developed based on different genetic or statistical assumptions. However, no single method is optimal for all traits and, for many traits, the putative single nucleotide polymorphisms (SNPs) that are detected by the different methods do not entirely overlap due to the diversity of the genetic architecture of complex traits. Therefore, multi-tool-based GWAS strategies that combine different methods have been increasingly employed. To take this one step further, we propose an ensemble-like GWAS strategy (E-GWAS) that statistically integrates GWAS results from different single GWAS methods.

**Results:**

E-GWAS was compared with various single GWAS methods using simulated phenotype traits with different genetic architectures. E-GWAS performed stably across traits with different genetic architectures and effectively controlled the number of false positive genetic variants detected without decreasing the number of true positive variants. In addition, its performance could be further improved by using a bin-merged strategy and the addition of more distinct single GWAS methods. Our results show that the numbers of true and false positive SNPs detected by the E-GWAS strategy slightly increased and decreased, respectively, with increasing bin size and when the number and the diversity of individual GWAS methods that were integrated in E-GWAS increased, the latter being more effective than the bin-merged strategy. The E-GWAS strategy was also applied to a real dataset to study backfat thickness in a pig population, and 10 candidate genes related to this trait and expressed in adipose-associated tissues were identified.

**Conclusions:**

Using both simulated and real datasets, we show that E-GWAS is a reliable and robust strategy that effectively integrates the GWAS results of different methods and reduces the number of false positive SNPs without decreasing that of true positive SNPs.

**Supplementary Information:**

The online version contains supplementary material available at 10.1186/s12711-023-00820-3.

## Background

Genome-wide association studies (GWAS) have been widely used to detect genetic variants that influence complex traits in humans, animals, and plants [1–4] and contribute to deciphering the genetic architecture underlying target traits [5]. However, the detection of false positive variants has been an ever-lasting issue in the development of GWAS methods. False positive variants can be caused by non-causal associations, including population structure or unequal kinship among individuals [6, 7]. Different methods have been developed to address this issue. Initially, the general linear models (GLM) that were used treated population structure as a fixed effect but failed to decipher the cryptic kinship among individuals [8]. Subsequently, the mixed-effect linear model (MLM) incorporated population stratification as a fixed effect and individual relatedness as a random effect, which provides greater control over false positive variants [9]. Based on MLM, a series of GWAS methods has been developed, with the main focus on improving computational efficiency [10–12]. However, most of these methods are still based on the single-locus test, which may be inappropriate for complex traits that are controlled by major loci and polygenes, due to the intrinsic limitation of the model hypothesis [13]. Alternatively, multi-locus methods and Bayesian methods can provide better solutions for investigating complex traits. Multi-locus methods mainly include the multi-locus mixed model (MLMM) [14], the fixed and random model circulating probability unification (FarmCPU) method [15], the multi-locus random effect mixed linear model (mrMLM) [13], and the Bayesian-information and linkage-disequilibrium iteratively nested keyway (BLINK) method [16]. Representatives of the Bayesian methods include Bayesian regression models [17] and the Bayesian sparse linear mixed model (BSLMM) [18].

Although different GWAS methods have been widely used, no single method is ideal for all traits due to the diversity of their genetic architectures. For many traits, the putative single nucleotide polymorphisms (SNPs) that are detected by different GWAS methods do not completely overlap. As a result, multi-tool-based GWAS strategies that combine different methods have been increasingly employed. For example, Muhammad et al. [19] used four multi-locus and three single-locus GWAS models to study the genetic architecture of agricultural traits in wheat. Liu et al. [20] suggested that multiple GWAS methods, especially single-locus and multi-locus methods, can be mutually complementary for the identification of quantitative trait loci (QTL). Nida et al. [21] emphasized the drawbacks of employing multiple models for GWAS because some loci may be overlooked or marginalized in certain GWAS models in spite of their significant contribution to the trait of interest.

Previous studies have shown that the combination of different GWAS methods can improve the detection rate and statistical robustness of major QTL and the use of multiple statistical methods to detect major QTL in GWAS is becoming more common for complex traits. However, to date, most studies have simply conducted different GWAS methods separately and reported the results in parallel mode without proper statistical integration. Such treatment does not represent a real integration analysis and cannot effectively control the number of false and true positive variants detected.

The stacked ensemble method [22] represents a potential solution to the issue of integrating the outcomes from multiple single GWAS methods although, to date, it has not been used for this purpose. Previous studies have applied stacked ensemble methods in genomic prediction to improve prediction accuracy [23–25]. Recently, ensemble learning methods have been used to investigate gene–gene (environment) interactions and the prioritization of disease genes in GWAS [26, 27]. There are several types of ensemble learning methods, such as bagging [28], boosting [29], and stacking [22], among which the stacked ensemble method results in a higher prediction accuracy than each single base model by integrating the outputs of different base models into a meta model [22]. Thus, the stacked ensemble method provides an effective tool for integrating the results from different single GWAS methods. Based on this, in this study, we developed an ensemble-like strategy to integrate multiple GWAS results and named it E-GWAS (ensemble-like GWAS). Our main objective was to systematically confirm the effectiveness of E-GWAS by statistically integrating a set of GWAS results from different types of GWAS methods.

## Methods

### Real dataset

In this study, we used a pig dataset that included 4555 individuals and one backfat thickness trait. All pigs were genotyped with the Illumina PorcineSNP50 Bead Chip. We excluded SNPs that had a minor allele frequency lower than 1%, a missing position, and/or a call rate lower than 95%. After filtering, 41,078 SNPs remained for further analysis. Missing genotypes were imputed by the Beagle 5.0 software [30].

### Simulated datasets

We generated simulated data based on the observed genotypes of the pig population in the real dataset. Phenotypes were simulated by the simulated phenotype generation function in the Genome Association and Prediction Integrated (GAPIT) Tool [31], which randomly samples $m$ SNPs as quantitative trait nucleotides (QTN) and generates the effect of each QTN ($\beta$) from a standard normal distribution or from a geometric distribution. Subsequently, it calculates the genetic value of each individual as $g=\sum_{j}^{m}{x}_{j}{\beta }_{j}$, where $x$ is coded as 0, 1, and 2 for genotypes *aa*, *Aa*, and *AA*, respectively. Finally, it generates the residual effects ($e$) from $N(0,var(g)(1-{h}^{2})/{h}^{2})$ and calculates the simulated phenotype as $y=g+e$.

We used different values for trait heritability (${h}^{2}$ = 0.2, 0.6, and 0.8) and for the number of QTN ($m$ = 20, 100, 500, and 1000). The effect of each QTN ($\beta$) was generated from a normal distribution (for $m$ = 20 and 100) or from a geometric distribution (for $m$ = 500 and 1000). For each setting, we repeated the simulation 100 times, randomizing the positions of the QTN in each replicate.

### Workflow of the E-GWAS strategy

**Fig. 1:**
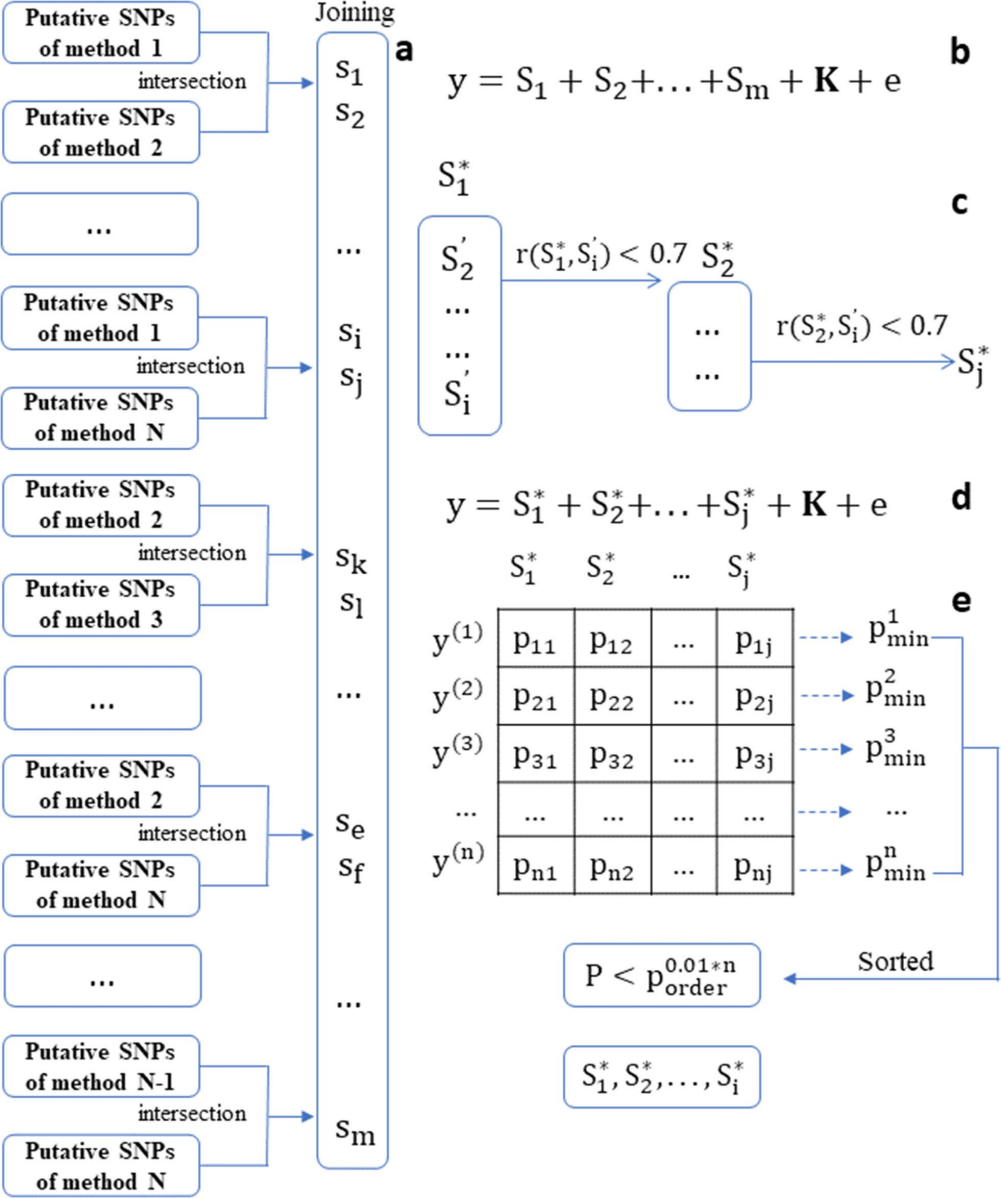
The procedure of E-GWAS. No single method can be entirely optimal for all traits due to the diversity of the genetic architectures of complex traits. For many traits, the putative SNPs detected by different methods do not completely overlap. Our proposed E-GWAS strategy integrates different GWAS methods to adapt different traits through a three-step procedure. First, we identify the overlapping SNPs between lists of SNPs for each pair of N GWAS methods and combine them to obtain a preliminary combined list with m SNPs. Because some putative SNPs identified by the different methods might be close to each other, a within-bin merged method can be used to expand the size of the intersection windows (i.e., 10 and 50 kb) (**a**). Then, the m SNPs are simultaneously integrated into a mixed-effect linear model as fixed effects, and their p-values are calculated (**b**). Among the m putative SNPs, SNPs with a Pearson correlation coefficient of their genotypes greater than 0.7 are clustered together and the j remaining SNPs are retained, those with the lowest p value in each of the j clusters (**c**). The j SNPs are again fitted in a mixed-effect linear model (**d**). The permutation test method is used to define the threshold for the j p-values corresponding to the fitted SNPs. The phenotype (y) is shuffled n times and the p-values of the j SNPs are re-calculated. Then, the 0.01-quantile of the n minima of the j p-values in n times is defined as the threshold (**e**). Finally, i SNPs with p-values below the threshold are retained

The E-GWAS strategy relies on the stacked ensemble method [22] by constructing the meta-processor for integrating the GWAS results from different single GWAS methods. Specifically, the E-GWAS strategy consists of three steps, as outlined below and visualized in Fig. 1, which aim at selecting putative SNPs that are truly associated with target traits based on the results of different GWAS methods.

(i) GWAS step. Five tools that are known to have high statistical power were chosen for separate GWAS, including one Bayesian method (BSLMM) and four multi-locus linear models (MLMM, mrMLM, FarmCPU, and BLINK). SNPs with Bonferroni-corrected p-values lower than the threshold ($\mathrm{\alpha }/\mathrm{M}$, $\mathrm{\alpha }$ = 0.01, where $\mathrm{M}$ is the number of SNPs) were defined as putative SNPs. The results of each GWAS were saved as lists of SNPs.

(ii) Intersection-joining step. We combined the overlapping SNPs between lists (intersections) from each pair of GWAS methods and joined together 10 intersections to obtain a preliminary combined list with $\mathrm{m}$ SNPs. Considering that some putative SNPs from different methods might be close to each other, a within-bin merged method was used to expand the intersection ranges (i.e., 10 and 50 kb). To avoid multicollinearity, the m SNPs are simultaneously integrated into a mixed-effect linear model as fixed effects, kinship among individuals as a random effect, and their p-values are calculated based on this model (Fig. 1b). Among the m putative SNPs, SNPs with a Pearson correlation coefficient of their genotypes greater 0.7 are clustered together and the j remaining SNPs are retained, those with the lowest p value in each of the j clusters (Fig. 1c).

(iii) Meta-processor step. To further control the number of false positive SNPs, the $\mathrm{j}$ SNPs were simultaneously integrated into a mixed-effect linear model again as fixed effects, and the $\mathrm{j}$ p-values corresponding to SNPs from the preliminary combined list were re-estimated together. We used the permutation test method to define the threshold for the $\mathrm{j}$ p-values corresponding to SNPs from the preliminary combined list. A null hypothesis of no association between SNP and trait was established. Let ${\mathbf{P}}_{0}$ denote any probability under the null hypothesis. The distribution under ${\mathbf{P}}_{0}$ of the $\mathrm{j}$ p-values was estimated by the following resampling procedure:

1. : Set $$\mathrm{n}=1$$
2. : Generate permutated phenotypes ${\mathbf{y}}_{\mathbf{n}}$ by random shuffling
3. : Apply a GLM by taking ${\mathbf{y}}_{\mathbf{n}}$ as the phenotypes to compute the $\mathrm{j}$ p-values
4. : Store the smallest of the $\mathrm{j}$ computed p-values as ${\mathrm{p}}_{\mathrm{min}}^{(\mathrm{n})}$
5. : Let $\mathrm{n}=\mathrm{n}+1$, repeat steps (2) to (4) 10,000 times
6. : Order the ${\mathbf{p}}_{\mathrm{min}}^{(\mathrm{n})}$, and return the vector ${\mathbf{p}}_{\mathrm{ordered}}$ as ${\mathbf{P}}_{0}$

Finally, the 0.01-quantile of the smallest of these k p-values (${p}_{ordered}^{0.01*10000}$) was defined as the threshold.

## Results

### E-GWAS outperformed all single methods tested in analysis of the simulated traits

**Fig. 2:**
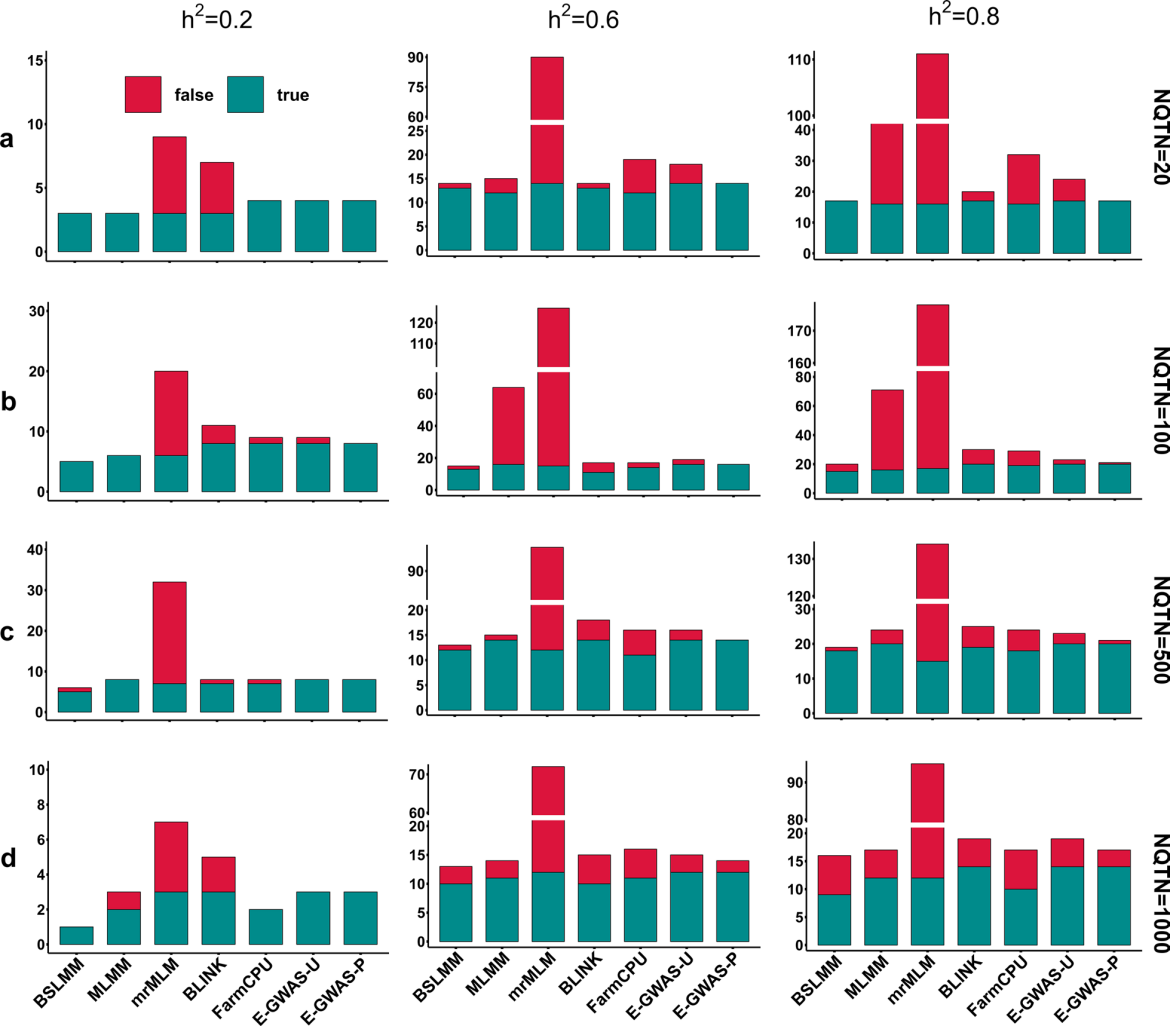
Comparison between E-GWAS and BSLMM, MLMM, mrMLM, BLINK, and FarmCPU using simulated phenotypes. Three levels of heritability were used in the simulations: 0.2 (left), 0.6 (middle), and 0.8 (right). Four gradients of QTN numbers were set for each level of heritability: 20 (**a**), 100 (**b**), 500 (**c**), and 1000 (**d**). The number of true QTN (crimson) and the number of false QTN (dark cyan) were recorded. For the E-GWAS-U, the permutation test was not used and for the E-GWAS-P, it was used. As the heritability increased, more true QTN were detected by the different methods and the E-GWAS retained a large number of true QTN. After the permutation test, compared with the single methods, E-GWAS resulted in a smaller number of false positives in each simulated scenario

We compared the performances of different methods based on simulated traits. Figure 2 displays the numbers of detected SNPs, true QTN, and false QTN obtained with the different methods under various simulated scenarios. Before running the permutation test, the number of true QTN obtained with E-GWAS was comparable to that of the five other single methods, and the rate of false positive variants detected in E-GWAS was higher than that of BSLMM, MLMM, and BLINK for several simulated traits. After running the permutation test, most of the true QTN were retained, and the rate of false positive variants detected in E-GWAS declined considerably, occasionally even reaching zero. These results show that the E-GWAS strategy provides effective control over the number of false positive variants without decreasing the number of true positive variants and outperforms the five others single GWAS methods.

**Fig. 3:**
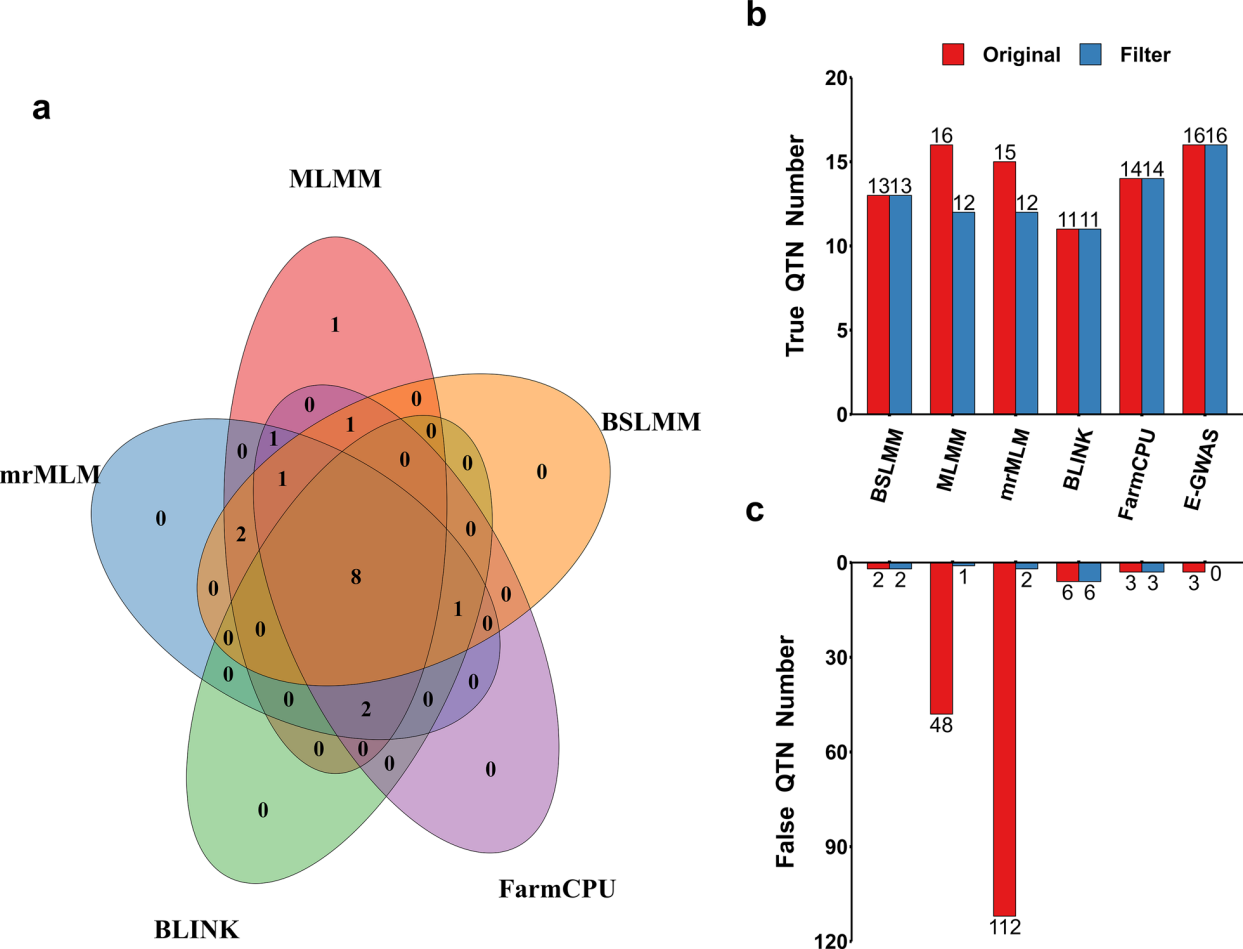
Performance of different methods before and after the permutation test. E-GWAS and five other methods including BSLMM, MLMM, mrMLM, BLINK, and FarmCPU were used to analyze a simulated phenotype (simulated trait with a heritability of 0.6 controlled by 100 QTN). **a** The Venn Diagram shows the overlapping of the true QTN detected by these methods. MLMM specifically detected one true QTN. The bar graphs in **b**, **c** separately exhibit the difference in the number of true and false QTN between single methods and E-GWAS before and after running the permutation test. Red and blue colors represent the number of QTN before and after filtering by the permutation test

Due to the differences between the various GWAS methods and to the complexity of the genetic architecture of complex traits, the QTN that were unique to a particular method were omitted in the intersection-joining step of E-GWAS (Fig. 3a). For comparison, we also ran permutation tests to filter the SNPs that were detected by BSLMM, MLMM, mrMLM, BLINK, and FarmCPU under one of the simulated scenarios, i.e. a simulated trait with a heritability of 0.6 controlled by 100 QTN. The p-values for the same SNP differed between methods. After running the permutation test, the numbers of true and pseudo QTN obtained by the different methods (except BSLMM, BLINK and FarmCPU) showed various degrees of reduction (Fig. 3b, c). Overall, compared with the five single GWAS methods, E-GWAS provided tight control over the rate of false positive variants and retained a large number of true QTN.

### E-GWAS stably controlled the rate of false positive variants across scenarios for traits with different genetic architectures

**Fig. 4:**
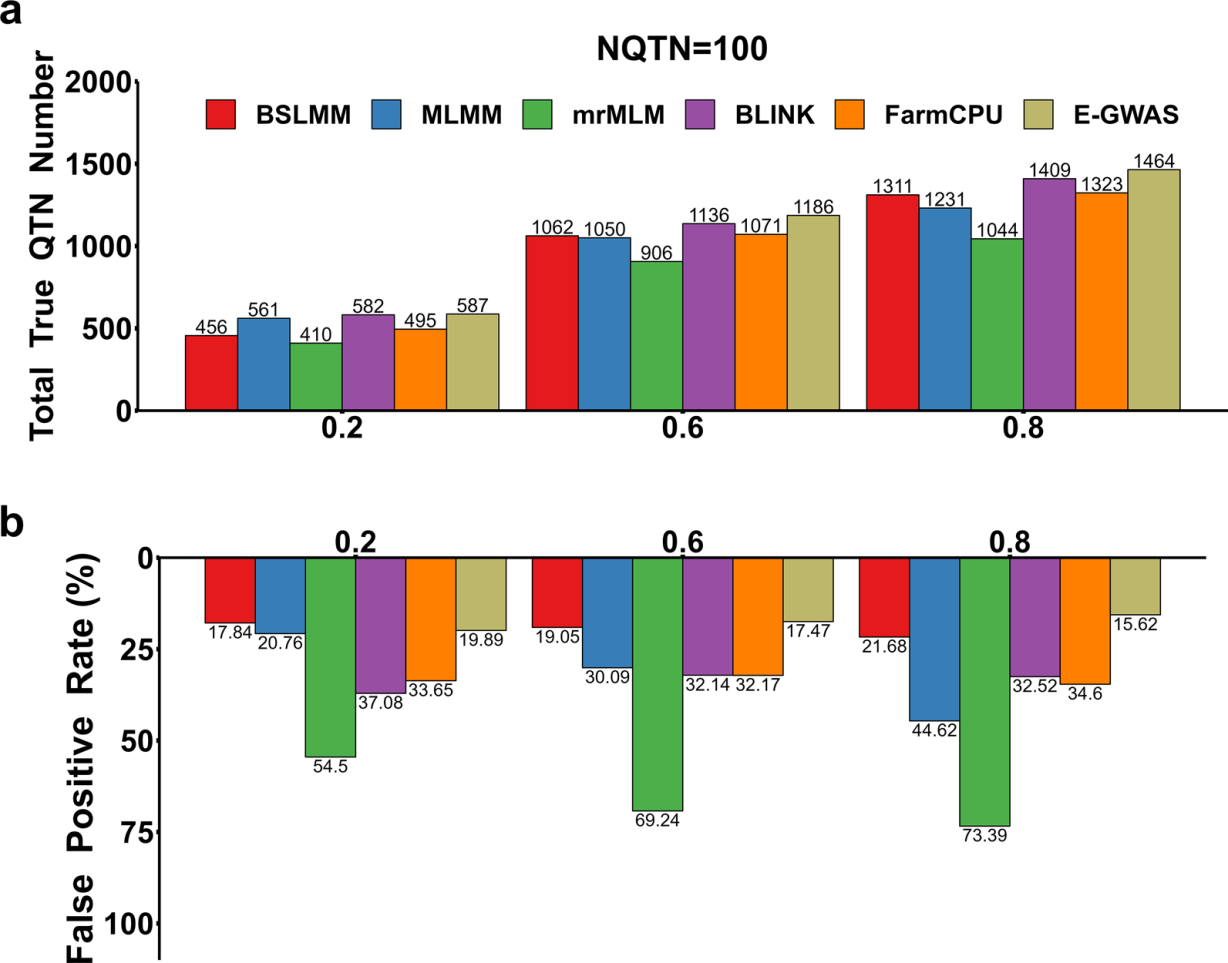
Comparison between E-GWAS and BSLMM, MLMM, mrMLM, BLINK, and FarmCPU under different heritability levels. **a** Detected true QTN numbers and **b** False positive rates. The comparisons were conducted with 100 replicates. The numbers of true and false positives among 100 replicates were recorded. The simulated phenotype was controlled by 100 QTN with different heritabilities, low (0.2), moderate (0.6), and high (0.8). The column represents the sum of the true QTN **a** or the average of false positive rate **b** observed from 100 simulations

**Fig. 5:**
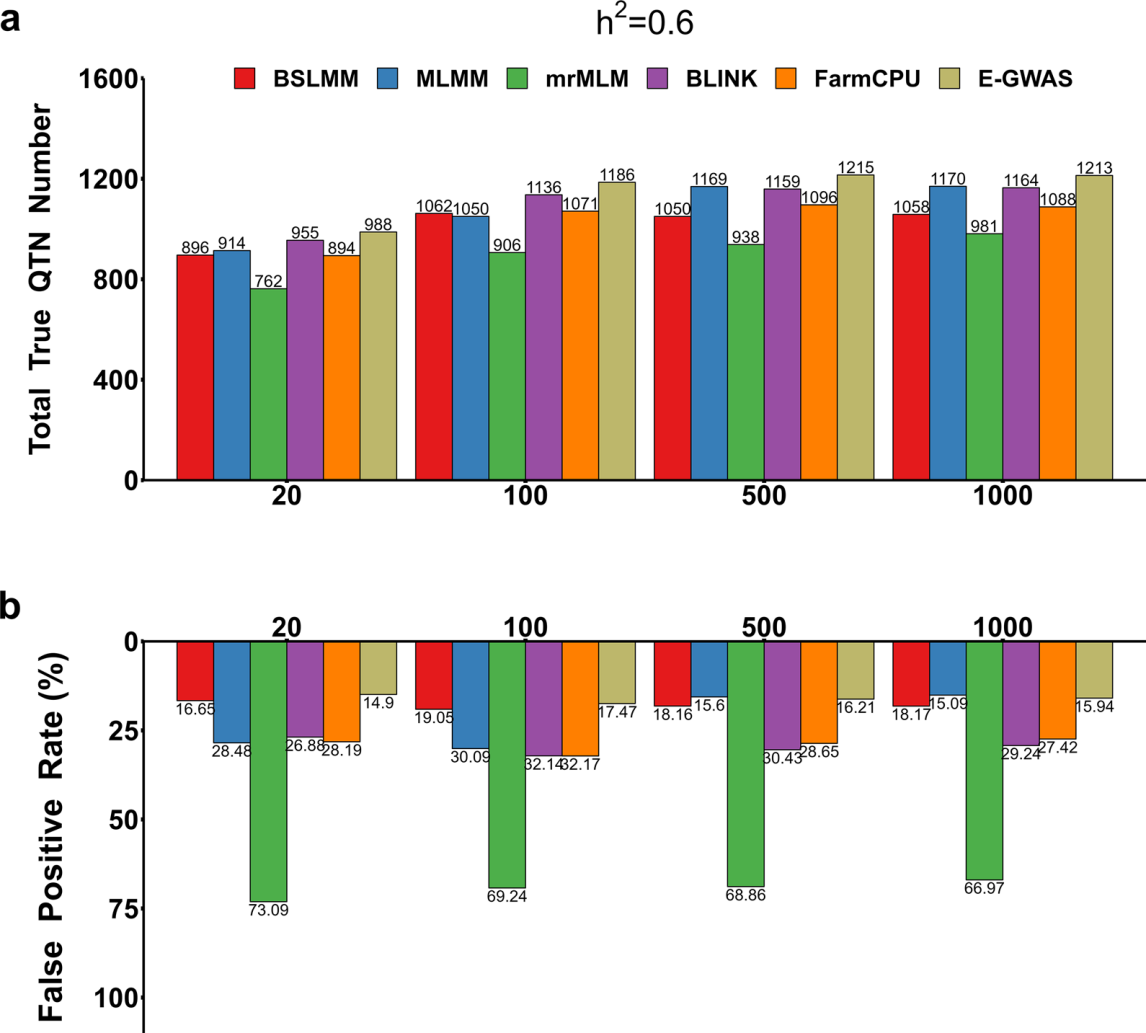
Comparisons between E-GWAS and BSLMM, MLMM, mrMLM, BLINK, and FarmCPU under different numbers of QTN. **a** Detected true QTN numbers and **b** False positive rates. The comparisons were conducted with 100 replicates. The numbers of true and false positives among 100 replicates were recorded. The simulated phenotypes had a heritability of 0.6 and were controlled by different numbers of QTN (20, 100, 500, and 1000). The column represents the sum of the true QTN **a** or the average of false positive rate **b** observed from 100 simulations

To verify the stability of E-GWAS across various scenarios, the abilities of E-GWAS, BSLMM, MLMM, mrMLM, BLINK, and FarmCPU in controlling the number of true and false positive variants were compared for traits with different genetic architectures across 100 replicates. The numbers of true and false positive variants detected in each scenario were recorded. Figures 4a and 5a show the number of true QTN detected by each method, which increased with increasing trait heritability, but E-GWAS outperformed all five individual GWAS methods across all scenarios with varying genetic architectures (Figs. 4a and 5a) and (see Additional file 1: Fig. S1a and Additional file 2: Fig. S2a).

Figures 4b and 5b show the rates of false positive variants detected by the different methods. Compared with E-GWAS, BSLMM had a higher rate of false positive variants for all simulated phenotypes (Figs. 4b and 5b) and (see Additional file 1: Fig. S1b and Additional file 2: Fig. S2b), except for phenotypes with a low heritability (0.2) (Fig. 4b) and (see Additional file 2: Fig. S2b). The rate of false positive variants detected by MLMM clearly fluctuated across scenarios for traits with different heritabilities and different numbers of true QTN (Fig. 5b) and (see Additional file 2: Fig. S2b). For the simulated phenotypes with a low (0.2) and moderate (0.6) heritability that were controlled by 500 or 1000 QTN, the rate of false positive variants detected was slightly lower for MLMM than for E-GWAS but MLMM had a smaller number of true positive variants (Fig. 5b) and (see Additional file 1: Fig. S1b and Additional file 2: Fig. S2b). For the other simulated phenotypes, the rate of false positive variants detected was higher for MLMM than for E-GWAS (Figs. 4b and 5b) and (see Additional file 1: Fig. S1b and Additional file 2: Fig. S2b). BLINK, FarmCPU, and mrMLM performed stably but were not optimal in terms of controlling the number of false positive variants across scenarios for traits with different genetic architectures. As expected, through the integration of the five methods, our proposed E-GWAS performed stably and was superior in controlling the number of false positive variants.

**Fig. 6:**
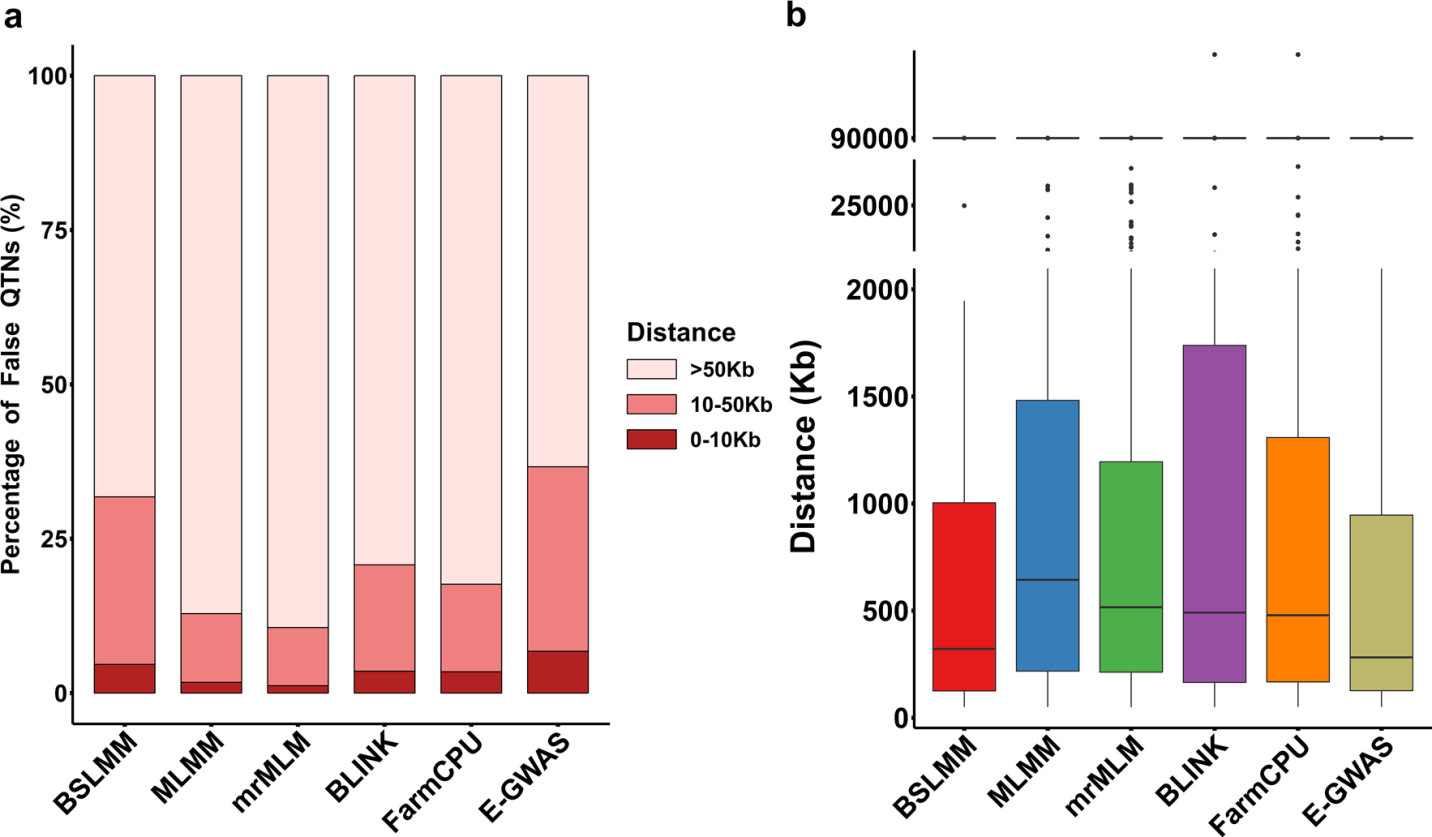
Distributions of the distances of putative SNPs to true QTN obtained with E-GWAS, BSLMM, MLMM, mrMLM, BLINK, and FarmCPU. We set two windows of 10,000, and 50,000 bp on either side of the true QTN. The simulated phenotypes had a heritability of 0.6 and were controlled by 100 QTN. The comparisons were conducted with 100 replicates. **a** Comparison of the proportions of putative SNPs that were located within each 10,000 and 50,000 bp window found between E-GWAS, BSLMM, MLMM, mrMLM, BLINK, and FarmCPU. **b** The distributions of the distances of the putative SNPs outside the 10,000, and 50,000 bp windows

Putative SNPs can correspond exactly to true QTN, but it is also common that putative SNPs are in the vicinity of true QTN due to linkage disequilibrium (LD). Therefore, two windows of 10,000, and 50,000 bp on either side of the true QTN were set. The numbers of putative SNPs present in each of these windows were compared for E-GWAS, BSLMM, MLMM, mrMLM, BLINK, and FarmCPU (Fig. 6a). Compared to the other methods, E-GWAS had a higher proportion of putative SNPs present in the flanking regions of either 10,000 or 50,000 bp. In addition, the putative SNPs outside of these windows were closer to true QTN (Fig. 6b). These results show that E-GWAS can lead to the identification of a larger number of putative SNPs associated with traits, while excluding some SNPs that result in false positives in individual GWAS methods. In the other scenarios, the proportions of putative SNPs present in the 10,000 and 50,000 bp windows were inversely proportional to the rate of false positives (see Additional file 3: Fig. S3). Overall, E-GWAS performed stably across scenarios for traits with different genetic architectures and provided effective control over the rate of false positives without decreasing the number of true positives.

### Applying a within-bin merged strategy improved the performance of E-GWAS

**Fig. 7:**
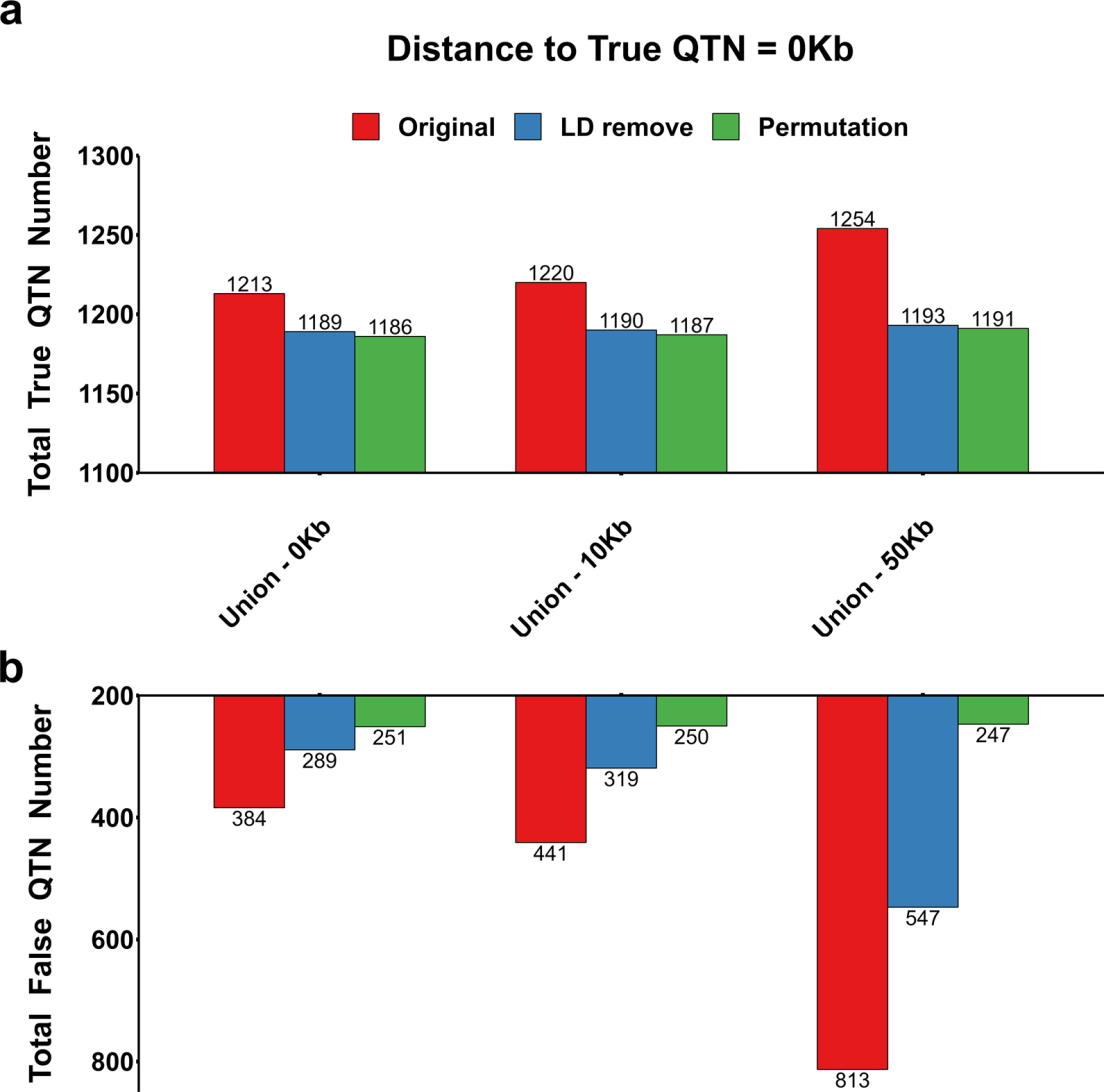
Performance of E-GWAS under different bin sizes. **a** Numbers of true QTN detected and **b** numbers of false QTN detected. We compared the performance of E-GWAS across three bin sizes: 0, 10 and 50 kb. The simulated phenotype had a heritability of 0.6 and was controlled by 100 QTN. The comparisons were conducted with 100 replicates. The numbers of true and false positives among 100 replicates were recorded. The preliminary combined SNP list, the SNP list after elimination multicollinearity among SNPs, and the list of remaining SNPs after the permutation test are represented in red, blue, and green

In E-GWAS, the intersection-joining step took only the putative overlapping SNPs with the same physical position from each pair of methods in account. However, we found that some putative SNPs that were detected by the different methods were close to each other, and also close to the true QTN, which could be attributed to the LD between the putative SNPs. These SNPs were removed in the intersection-joining step, which might weaken the performance of E-GWAS to a certain extent. Inspired by the bin method [15, 32], the putative SNPs within the same bins were retained in the intersection-joining step. Two bin sizes of 10 and 50 kb were set in the intersection-joining step. Figure 7 displays the performance of E-GWAS for these two bin sizes. The bin method in the intersection-joining step improved the ability of E-GWAS to detect true QTN but also resulted in a looser control over false positives. True QTN were partially removed in the redundancy reduction and the proportion of removed QTN increased with increasing bin size (Fig. 7a). In addition, the number of false QTN was still relatively large and increased with increasing bin size. After running the permutation test, most of the true QTN were retained and most of the false QTN were removed. We also considered scenarios in which a putative SNP was present in 10,000 and 50,000 bp windows on either side of a true QTN but the results were similar (see Additional file 4: Fig. S4), i.e. the numbers of true and false positives detected by E-GWAS slightly increased and decreased, respectively, with increasing bin size.

### Addition of single GWAS methods enhanced the performance of E-GWAS

Referring to the stacked ensemble method, the performance of E-GWAS could be influenced by the number and the diversity of the single GWAS methods integrated. We classified GWAS methods into three types based on differences in their genetic or statistical assumptions: single-locus methods, multi-locus methods, and Bayesian methods. To compare the performance of E-GWAS in different scenarios, we added different types or numbers of GWAS methods to four multi-locus GWAS methods (MLMM, FarmCPU, mrMLM, and BLINK).

**Fig. 8:**
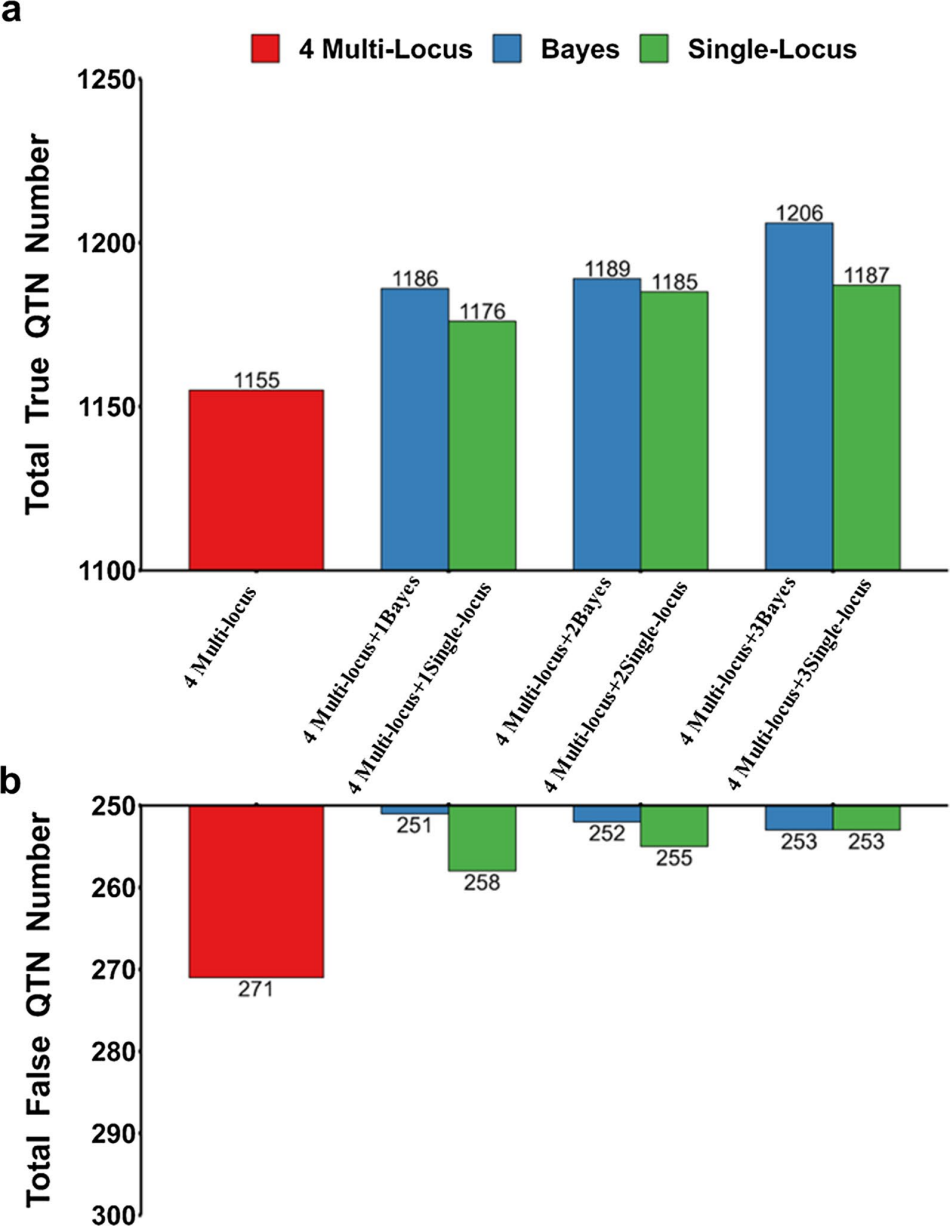
Comparison between E-GWAS when integrating different numbers of methods. **a** Numbers of true QTN detected and **b** numbers of false QTN detected. The simulated phenotype had a heritability of 0.6 and was controlled by 100 QTN. Based on the integration of four multi-locus methods (MLMM, mrMLM, BLINK, and FarmCPU), we compared the performance of E-GWAS by adding one to three single-locus methods (EMMAX, GEMMA, and FastGWA) in sequence, and adding one to three Bayesian methods (BSLMM, BayesB, and BayesCπ) in sequence. The comparisons were conducted with 100 replicates. The numbers of true and false positives across 100 replicates were recorded. E-GWAS that integrated four multi-locus methods, E-GWAS that integrated four multi-locus methods and three single-locus methods, and E-GWAS that integrated four multi-locus methods and three Bayesian methods are represented in red, green, and blue, respectively

Figure 8 shows the performances of E-GWAS that integrate different numbers of GWAS methods but of the same type. We added one to three single-locus GWAS methods in sequence, including efficient mixed-model association eXpedited (EMMAX) [33], genome-wide efficient mixed-model association (GEMMA) [34], and MLM-based genome-wide association (FastGWA) [12]. Similarly, we added one to three Bayesian methods in sequence, including BSLMM, BayesB, and BayesCπ. The performance of E-GWAS improved increasingly with one to three single-locus methods (EMMAX, GEMMA, and FastGWA) added, but the improvement in performance was smaller than that obtained by adding one to three Bayesian methods (BSLMM, BayesB, and BayesCπ) (Fig. 8). These results show that when GWAS methods of the same type are added, the Bayesian methods resulted in greater improvement of the performance of E-GWAS.

**Fig. 9:**
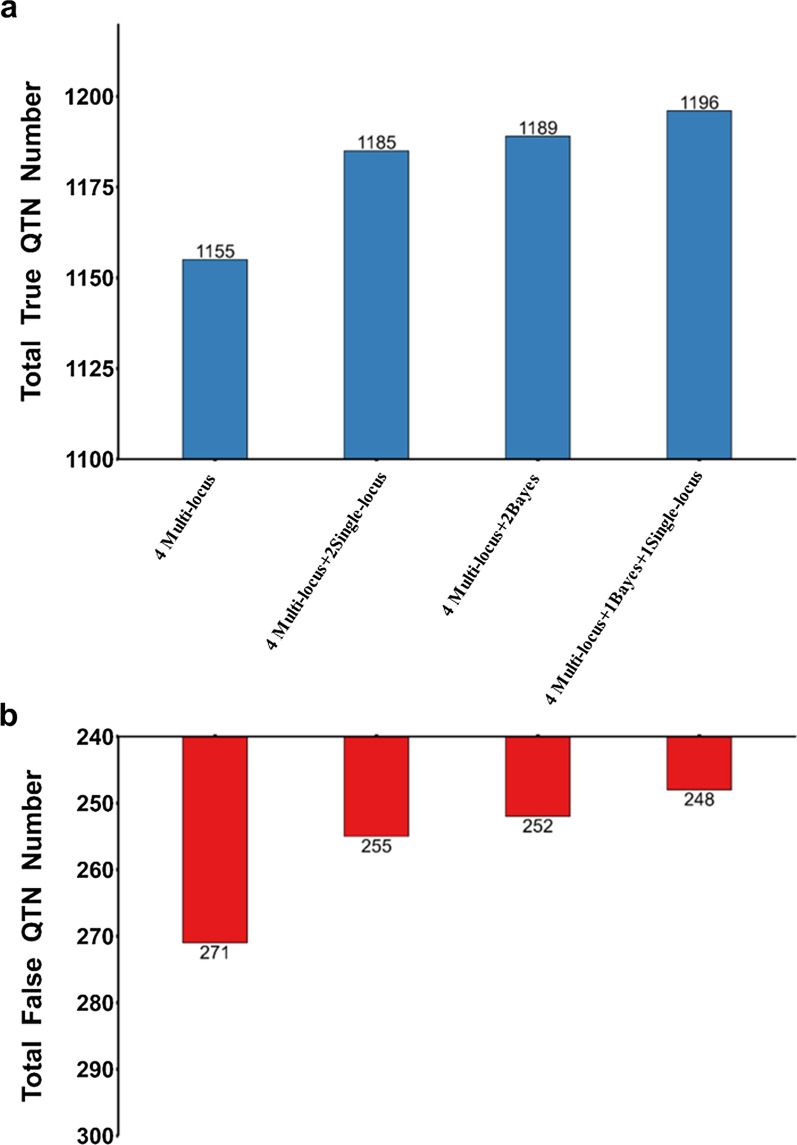
Comparison between E-GWAS when integrating different types of methods. **a** Numbers of true QTN detected and **b** numbers of false QTN detected. The simulated phenotype had a heritability of 0.6 and was controlled by 100 QTN. Based on the integration of four multi-locus methods (MLMM, mrMLM, BLINK, and FarmCPU), we compared the performance of E-GWAS by adding two single-locus methods (GEMMA and FastGWA), adding two Bayesian methods (BSLMM and Bayes B), and adding one single-locus method (FastGWA) and one Bayesian method (BSLMM). The comparisons were conducted with 100 replicates. The numbers of true and false positives among 100 replicates were recorded

Figure 9 shows the performance of E-GWAS when the same number of methods is added but of different types. Adding one single-locus method (FastGWA) and one Bayesian method (BSLMM) was more effective in improving the performance of E-GWAS than adding two Bayesian methods (BSLMM and Bayes B) or two single-locus GWAS methods (GEMMA and FastGWA). These results show that when the same number of GWAS methods is integrated, the performance of E-GWAS improves increasingly as more types of methods are used.

### Application of E-GWAS to a pig dataset produced reliable results

To assess the performance of the E-GWAS strategy on a real dataset, we analyzed backfat thickness for a pig population (n = 4555 individuals and m = 41,078 SNPs after quality control). The phenotypic distribution of backfat thickness nearly followed a normal distribution (see Additional file 5: Fig. S5). GWAS for backfat thickness were conducted by MLMM, mrMLM, FarmCPU, BLINK, BSLMM, BayesB, BayesCπ, and FastGWA. Bonferroni correction was applied to adjust the p values. Respectively two, nine, six, six, one, one, one, and eight putative SNPs (p-value < 2.12E−07) were detected by these eight single GWAS methods.

Subsequently, the overlapping putative SNPs detected by every pair of GWAS methods were merged into a combined set, which retained four SNPs after running the permutation test. The same four SNPs were also obtained when the bin size was set to 10 and 50 kb in the intersection-joining step. These four SNPs were located on chromosomes 1, 7, 9, and 13. The 1-Mb genomic regions on either side of the obtained SNPs were considered as candidate regions. Linkage disequilibrium analyses of the four candidate regions were conducted with LD block plots (see Additional file 6: Fig. S6). After genome annotation, the genes related to adipose tissue function within these candidate regions were considered as candidate genes, resulting in 10 candidate genes in total. Information on the SNPs and corresponding candidate genes is provided in Table 1.

**Table 1:** Identified SNPs after running the permutation test for backfat thickness

SNP ID	Chr	Physical position (bp)	Candidate genes
MC4R	1	160,773,442	*LMAN1*, *PMAIP1*, *MC4R*, *SEC11C*
DRGA0007123	7	9,947,893	*RANBP9*, *SIRT5*, *MCUR1*, *CD83*
WU_10.2_9_3423450	9	2,722,389	*SMPD1*
WU_10.2_13_10321624	13	9,051,813	*UBE2E2*

**Fig. 10:**
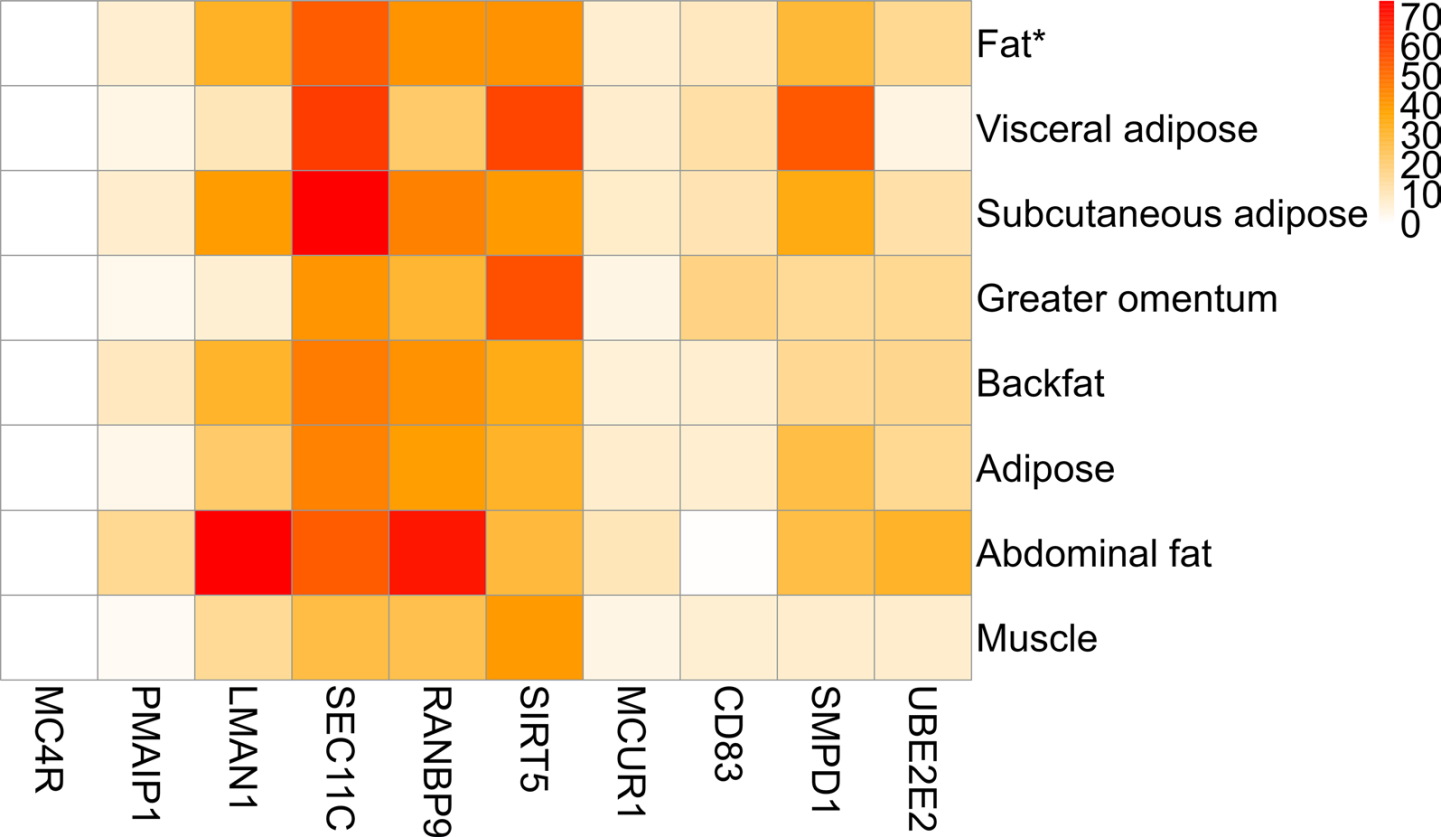
Expression profiles of 10 candidate genes in fat-related tissues. The horizontal axis indicates gene symbols, and the vertical axis denotes the names of the adipose-related tissues. The color bar represents expression abundance. The unit of expression value is transcripts per million (TPM). The gene expression fat value is the average of the adipose tissues in different parts of the body

To validate the potential roles of these candidate genes, the ISWINE database [35] (http://iswine.iomics.pro) was used to construct their tissue-specific expression profile in fat-related tissues. The target tissues included visceral adipose, subcutaneous adipose, greater omentum, backfat, adipose, abdominal fat, and musculature. Gene expressions in the target tissues were visualized by a heat map (Fig. 10), where the *SEC11C, LMAN1, RANBP9,* and *SIRT5* genes had a higher expression abundance in fat-related tissues and musculature, whereas the *MC4R* gene had an extremely low abundance. The expression profile of the candidate genes showed that they were expressed in fat-related tissues, which indicates that they may influence backfat thickness and that the results provided by the E-GWAS using a real dataset are reliable.

## Discussion

To date, GWAS have been widely used for the genetic dissection of complex traits. However, the rate of false positive variants is an ever-lasting problem in GWAS. Several GWAS methods have been proposed to solve this issue, including the multi-tool-based GWAS strategy, and its use is increasingly reported in the literature. Previous studies have shown that when several GWAS methods are used to analyze the same dataset, the putative SNPs that are identified by different approaches tend to be more reliable [36, 37]. Thus, using several GWAS methods, especially different types of GWAS methods, tends to be more effective to investigate the genetic architecture of target traits. However, not all QTN can be identified by each statistical method, and since different GWAS methods are usually based on different genetic or statistical assumptions, interpretation of the results can be difficult. The E-GWAS strategy provides a possible solution to overcome this difficulty.

### Considerations for the removal of redundant putative SNPs in E-GWAS

The advantage of the intersection-joining step in E-GWAS is that it takes the overlapping putative SNPs with the same physical position from each pair of methods into account. Our results show that the putative SNPs detected by E-GWAS were closer to true QTN than those by the other methods. This can be attributed to the fact that some of the putative SNPs detected by the different methods are in the vicinity of true QTN, but the SNPs that result in false positives differ between methods. Therefore, putative SNPs related to the trait under study can be obtained by the intersection-joining step, while the SNPs that result in false positives in each method can be excluded. In addition, we found that using a within-bin merged method to expand the size of the intersection windows can improve the number of true positives. Considering that the putative SNPs that are detected within the same region by different methods may differ but may be close to each other, E-GWAS uses a within-bin merged method to expand the size of the intersection windows and to merge the signals, some of which are true QTN.

In the process of removing redundant putative SNPs, the p-values of the putative SNPs in the combined list were calculated using the mixed-effect linear model. If two putative SNPs had Pearson correlation coefficients above a threshold (e.g., 0.7), the less significant putative SNP was removed. When the p-values of the putative SNPs were calculated, we found that the significance level of a true QTN tended to decrease when some putative SNPs were highly associated with the QTN. Thus, when redundant putative SNPs are removed, some true QTN might be erroneously removed. In addition, a small proportion of the true QTN was removed based on the permutation test, possibly because the effect of some true QTN is less significant. Although some true QTN were lost by removing some redundant putative SNPs and by the permutation test, many false QTN were removed and most of the true QTN were retained.

### Effect of the number and diversity of the integrated methods on the performance of E-GWAS

The E-GWAS strategy relies on the stacked ensemble method, which can yield better results when the base models are heterogeneous [38, 39]. It has been reported that the single-locus method performs poorly for complex traits [13, 14, 40], while the multi-locus methods and Bayesian methods show better performance for such traits [17, 18]. Following the stacked ensemble idea, the single-locus method was regarded as the weak learner and the multi-locus and Bayesian methods, as the strong learner. Studies reported in the literature have shown that the performance of the stacked ensemble method depends on the number and the diversity of the base learners, both weak and strong [41, 42]. Similarly, the performance of E-GWAS can be influenced by the number and the diversity of the GWAS methods that are integrated.

The performance of E-GWAS was slightly improved by adding single-locus methods, but this improvement in performance was smaller than that obtained by adding a Bayesian method. The reason for this difference might be that the genetic or statistical assumptions of the Bayesian methods are more appropriate for complex traits. The simultaneous addition of single-locus methods and Bayesian methods resulted in greater improvement of the E-GWAS performance than the addition of single-locus methods or Bayesian methods alone. As the diversity of or the heterogeneity between the integrated GWAS methods increases, more traits with different genetic architectures can be covered. This is where the stacked ensemble strategy is effective. In principle, adding more different types of GWAS methods can cover more complex genetic architectures of traits. However, integrating more GWAS methods also increases the computational burden of the E-GWAS.

### Considerations for improving the computational efficiency of E-GWAS

In ensemble methods, it is assumed that there is an ideal number of component learners and that accuracy will decrease if the number of learners is more or less than this number [43]. Likewise, the main factor that affects the performance of E-GWAS is the adaptability of the added GWAS methods to the genetic architecture of the target trait. Figures 8 and 9 show that the number of true and false positive QTN detected by E-GWAS increased and decreased, respectively, as more GWAS methods were integrated. Nevertheless, when these numbers given in Figs. 8 and 9 are divided by 100 (for considering an average gain per replicate), the gain in true QTN and the reduction in false QTN were less than 0.5 on average, which suggests that integrating more GWAS methods may not be appropriate for all phenotypes. Although our simulation results indicated that E-GWAS performed well when more Bayesian methods and multi-locus methods, and fewer single-locus methods were used, how to run E-GWAS efficiently is an interesting issue.

When the exact genetic architecture of the target trait is unknown, the composition and number of GWAS methods that should be integrated in the E-GWAS are largely determined by experience. When running E-GWAS on a big dataset, computing time should be a primary consideration. In the E-GWAS strategy, the time cost of the meta-processor step did not significantly differ when the number of GWAS methods increased since a relatively small number of SNPs were obtained in the intersection-joining step. Thus, the computing time of the whole E-GWAS process depended largely on the efficiency of the chosen GWAS methods. Previous studies have shown that the FarmCPU, BLINK, BSLMM, and FastGWA methods are computationally efficient for large datasets [12, 16, 18, 44], which indicates that they can be used for E-GWAS. To test computing time, we created a synthetic dataset by randomly duplicating a pig dataset (n = 4555 individuals and m = 41,078 SNPs after quality control). It took E-GWAS about 6 h to complete the analysis of the large synthetic dataset with 100,000 samples (R language platform, Intel Core i5 CPU 7500, 3.40 GHz, Memory 32.00G).

A sample size with sufficient statistical power is critical to the success of GWAS to detect causal variants for complex traits [45]. When conducting GWAS on moderate or small datasets, the differences in the results between GWAS methods may increase. For such datasets, we highly recommend using E-GWAS and adding more GWAS methods of different types to cover the genetic architecture of different target traits. For a reasonable amount of computing time, we suggest using MLMM, FarmCPU, mrMLM, BLINK, BSLMM, and FastGWA methods to accommodate the genetic architecture of complex traits.

### The reliability of E-GWAS on the real pig dataset

The putative SNPs detected by E-GWAS were reliable in the pig dataset. Using the pig dataset, based on the GWAS results of eight single methods for backfat thickness, four SNPs were eventually detected by E-GWAS and 10 candidate genes were identified after genome annotation. The *MC4R* gene is known to affect porcine backfat thickness [46, 47] and the *SIRT5* gene to regulate adipose formation in pigs, cattle, and mice [48–50]. The ran-binding protein 9 which is encoded by the *RANBP9* gene interacts with the androgen receptor [51] and SNPs in the *androgen receptor* gene have been shown to be associated with fatness in pigs [52]. The *SMPD1* gene has been reported to be up-regulated in the lipid biosynthetic process in pigs [53], and its expression level in human adipose tissue is higher in people who have a high level of liver fat [54].

Interestingly, the *SEC11C, MCUR1,* and *LMAN1* genes are known to be associated with the growth of pigs [55–57]. Previous studies have confirmed the negative correlation between fat-related traits and growth-related traits [58], which suggests that these genes may be associated with backfat thickness. Other candidate genes which have been reported in humans, for example, the *PMAIP1* and *UBE2E2* genes are involved in human obesity [59–61]. The *CD83* gene is known to contribute to T lymphocyte proliferation [62], and in humans, it has been shown that the T cells are actively regulated in the adipose tissue and contribute to obesity-induced inflammation [63, 64]. Because pigs and humans share many genetic and physiological traits, it is possible that these genes also have corresponding functions in pigs, but further investigation is needed.

## Conclusions

In this paper, we propose an E-GWAS strategy and compare it systematically with single GWAS methods. The effectiveness of E-GWAS was validated by using both simulated and real datasets. The simulations showed that E-GWAS significantly reduced the number of false positive variants and efficiently controlled the number of true positive variants across different genetic backgrounds. In the real dataset, the putative SNPs identified by E-GWAS were also proven to be reliable. Thus, E-GWAS provides a reliable and robust strategy that effectively integrates the GWAS results from different individual methods and reduces the number of false positives without decreasing that of true positives.

## Supplementary Information

**Additional file 1: Figure S1.** Comparison between E-GWAS strategy and BSLMM, MLMM, mrMLM, BLINK, or FarmCPU under different levels of heritability with 20, 500, and 1000 QTN, respectively. The comparisons were conducted between different simulated scenarios. The numbers of true and false positives among 100 replicates were recorded. Phenotypes were simulated based on the real genotypes of 4555 individuals and 41,078 SNPs with different numbers of QTN (m = 20, 500 and 1000) and different levels of heritability (h^2^ = 0.2, 0.6 and 0.8). Line charts **a**, **b** illustrate the numbers of true QTN detected and the rate of false positive variants, respectively. BSLMM, MLMM, mrMLM, BLINK, FarmCPU, and E-GWAS are represented by different colors, as shown in the legend.

**Additional file 2: Figure S2.** Comparison between E-GWAS strategy and BSLMM, MLMM, mrMLM, BLINK, or FarmCPU under different numbers of QTN with a heritability of 0.2 and 0.8. The comparisons were conducted between different simulated scenarios. The numbers of true and false positives among 100 replicates were recorded. Phenotypes were simulated based on the real genotypes of 4555 individuals and 41,078 SNPs with different numbers of QTN (m = 20, 100, 500 and 1000) and different levels of heritability (h^2^ = 0.2, 0.8). Line charts **a**, **b** illustrate the numbers of true QTN detected and the rate of false positive variants, respectively. BSLMM, MLMM, mrMLM, BLINK, FarmCPU, and E-GWAS are represented by different colors, as shown in the legend.

**Additional file 3: Figure S3.** Comparison of the proportions of false QTN that were located in the different windows on either side of the true QTN between the E-GWAS strategy and BSLMM, MLMM, mrMLM, BLINK, or FarmCPU for traits with different genetic architectures. Three heritabilities for the simulated phenotype were used: 0.2 (left), 0.6 (middle), and 0.8 (right). The four rows show (from top to bottom) the different numbers of simulated QTN: 20 (row 1), 100 (row 2), 500 (row 3), and 1000 (row 4). Dark red, light red, and pink represent the putative SNPs that are located in the 10,000 bp window, the putative SNPs that are located in the 50,000 bp window, and the putative SNPs that are located outside of these two windows, respectively.

**Additional file 4: Figure S4.** Performance of E-GWAS at different sizes of the bin. **a**, **b** Numbers of true QTN detected. **c**, **d** Numbers of false QTN detected. Two distances to true QTN were set to define whether the detected SNP was a true QTN: 10 kb (left) and 50 kb (right). We compared the performance of E-GWAS for three bin sizes: 0, 10 and 50 kb. The preliminary combined list of SNPs, the SNP list after elimination multicollinearity among SNPs, and the list of remaining SNPs after running the permutation test are represented in red, blue, and green, respectively.

**Additional file 5: Figure S5.** Phenotypic distribution for backfat thickness. The Kolmogorov–Smirnov test indicates that the phenotype followed normal distributions similarly (p-value = 5.14E−12).

**Additional file 6: Figure S6.** Linkage disequilibrium block plots of four SNPs from the E-GWAS result. Panels **a**–**d** show the LD within 1 Mb upstream and downstream of the MC4R, DRGA0007123, WU_10.2_9_3423450, and WU_10.2_13_10321624 SNPs, respectively. The color bar represents the degree of linkage (${\mathrm{D}}^{^{\prime}}$) between two SNPs.

## Data Availability

The pig data and simulated phenotypes analyzed during the current study are available in the 2022 figshare repository: https://doi.org/10.6084/m9.figshare.21130672.
