# Supplementary figures and images for: E-GWAS: an ensemble-like GWAS strategy that provides effective control over false positive rates without decreasing true positives
Source: Genet Sel Evol. 2023 Jul 5;55:46. doi: 10.1186/s12711-023-00820-3 (PMC10320972; doi:10.1186/s12711-023-00820-3)

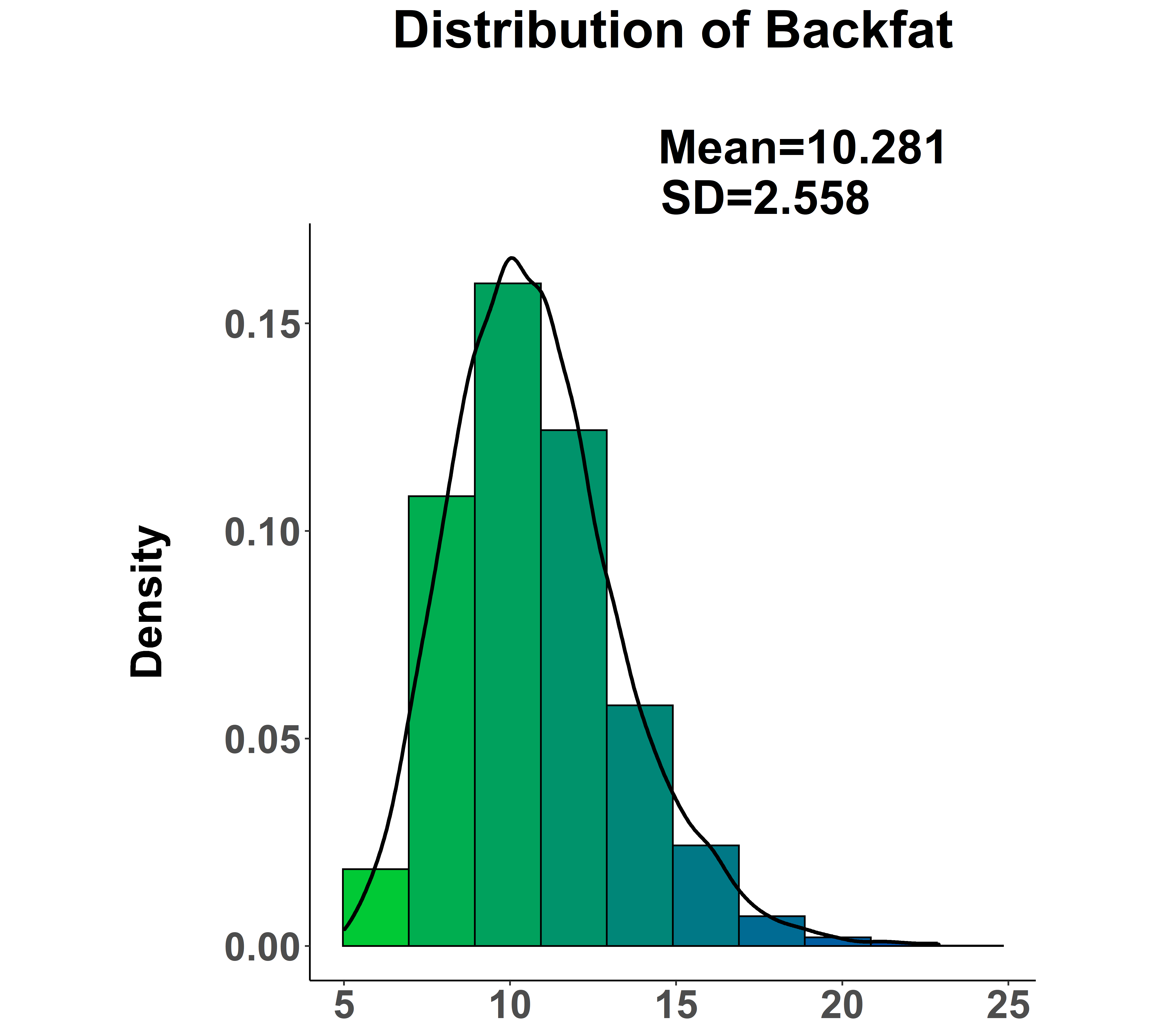

Supplement: Supplementary file 4 — Additional file 4: Figure S4. Performance of E-GWAS at different sizes of the bin. a, b Numbers of true QTN detected. c, d Numbers of false QTN detected. Two distances to true QTN were set to define whether the detected SNP was a true QTN: 10 kb (left) and 50 kb (right). We compared the performance of E-GWAS for three bin sizes: 0, 10 and 50 kb. The preliminary combined list of SNPs, the SNP list after elimination multicollinearity among SNPs, and the list of remaining SNPs after running the permutation test are represented in red, blue, and green, respectively. [file 12711_2023_820_MOESM4_ESM.png]

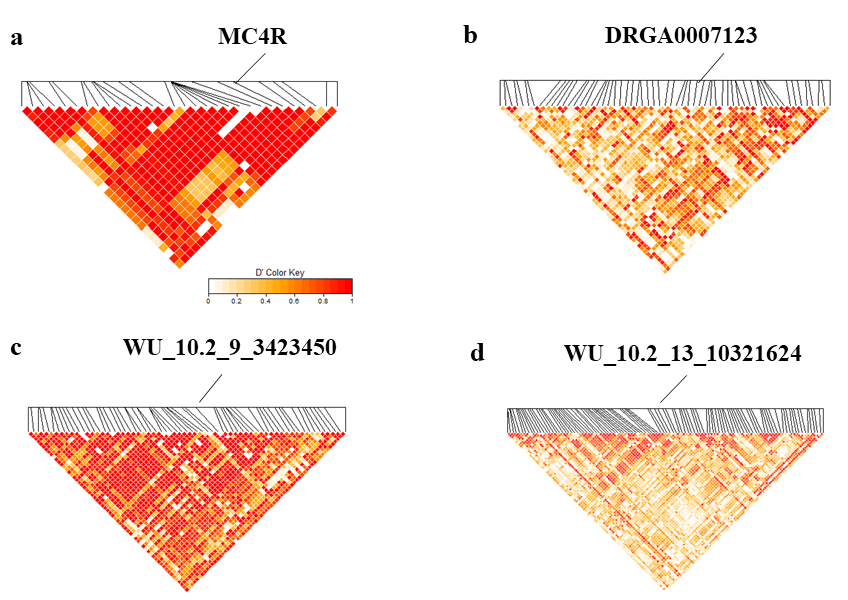

Supplement: Supplementary file 5 — Additional file 5: Figure S5. Phenotypic distribution for backfat thickness. The Kolmogorov–Smirnov test indicates that the phenotype followed normal distributions similarly (p-value = 5.14E−12). [file 12711_2023_820_MOESM5_ESM.png]
